# Resistance to DNA repair inhibitors in cancer

**DOI:** 10.1002/1878-0261.13224

**Published:** 2022-06-05

**Authors:** Joseph S. Baxter, Diana Zatreanu, Stephen J. Pettitt, Christopher J. Lord

**Affiliations:** ^1^ The CRUK Gene Function Laboratory and Breast Cancer Now Toby Robins Research Centre The Institute of Cancer Research London UK

**Keywords:** ATR, Cancer, DDR, PARP, PolQ, WRN

## Abstract

The DNA damage response (DDR) represents a complex network of proteins which detect and repair DNA damage, thereby maintaining the integrity of the genome and preventing the transmission of mutations and rearranged chromosomes to daughter cells. Faults in the DDR are a known driver and hallmark of cancer. Furthermore, inhibition of DDR enzymes can be used to treat the disease. This is exemplified by PARP inhibitors (PARPi) used to treat cancers with defects in the homologous recombination DDR pathway. A series of novel DDR targets are now also under pre‐clinical or clinical investigation, including inhibitors of ATR kinase, WRN helicase or the DNA polymerase/helicase Polθ (Pol‐Theta). Drug resistance is a common phenomenon that impairs the overall effectiveness of cancer treatments and there is already some understanding of how resistance to PARPi occurs. Here, we discuss how an understanding of PARPi resistance could inform how resistance to new drugs targeting the DDR emerges. We also discuss potential strategies that could limit the impact of these therapy resistance mechanisms in cancer.

## Graphical abstract

Drug resistance is a common phenomenon that impairs the overall effectiveness of cancer treatments including DNA repair inhibitors. How PARP inhibitor resistance emerges is partially understood. Here, we discuss how resistance to inhibitors of ATR kinase, WRN helicase or the DNA polymerase/helicase Polθ could emerge and how DNA repair inhibitor resistance could be targeted.

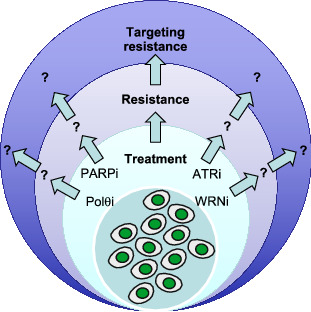

**Abbreviations**
*ATR*
: Ataxia telangiectasia and Rad3 related gene
*BRCA1*
: BRCA1 DNA repair‐associated gene
*BRCA2*
: BRCA2 DNA repair‐associated gene
ctDNA: circulating tumour DNA
DDR: DNA damage response network
HR: homologous recombination
MMR: mismatch DNA repair
NAD+: nicotinamide adenine dinucleotide
PAR: poly‐ADP‐ribose
*PARP1*
: poly‐(ADP‐ribose) polymerase 1 gene
PARPi: PARP inhibitor
*POLQ*
: DNA polymerase theta gene, protein known as Polθ
RS: replication stress
SWI/SNF: SWItch/sucrose non‐fermentable chromatin remodelling complex
TMEJ: theta‐mediated end joining
VEGF: vascular endothelial growth factor
WGR: protein domain containing tryptophan (W), glycine (G), arginine (R)
*WRN*
: Werner syndrome ATP‐dependent helicase gene
ZnF: Zinc Finger protein domain

## 1 Introduction

The DNA damage response (DDR) represents a complex network of proteins that detect and repair DNA. In doing so, the DDR maintains the integrity of the genome and prevents the transmission of mutations and rearranged chromosomes to daughter cells [1, 2, 3]. Consistent with this role, faults in the DDR (for example those caused by deleterious mutations in DNA repair associated tumour suppressor genes such as *BRCA1* and *BRCA2*) provide the mutagenic fuel that drives oncogenesis and are well described as drivers and hallmarks of cancer [4, 5, 6]. Apart from their driver effect in cancer, DDR defects in tumours also provide the basis for a number of therapeutic approaches. For example, platinum salt chemotherapy works in part by causing DNA inter‐ and intra‐strand crosslinks that breast or ovarian cancers with homologous recombination (HR) defects [7, 8] or lung cancers with nucleotide excision repair defects [9] are unable to effectively repair (Glossary). More recently, targeted agents that inhibit enzymes in the DDR have been developed as treatments in cancers with specific DDR defects. This is exemplified by PARPi used to treat breast, prostate, pancreatic or ovarian cancers with defects in the HR pathway, controlled by the tumour suppressors including (but not exclusive to) *BRCA1, BRCA2, PALB2, RAD51C* and *RAD51D* [10, 11, 12, 13]. For example, in gynaecological cancers, the PARPi olaparib is approved for use as a maintenance treatment for advanced cancer patients with deleterious or suspected germline or somatic *BRCA1/2*‐mutations who have shown a prior response to first‐line platinum‐based chemotherapy (a clinical indication that HR is defective) [14]. Olaparib is also used in gynaecological cancers as part of a combination maintenance treatment with the VEGF inhibitor bevacizumab, in patients who show either a complete or partial response to first‐line platinum‐based chemotherapy (Glossary), or those with defined HR deficiency (HRD) defined by a deleterious or suspected deleterious *BRCA1/2* mutation and/or an FDA‐approved diagnostic that estimates the presence of cancer‐associated genomic rearrangements normally associated with HRD [14]. Finally, olaparib is also used for the treatment of adult gynaecological cancer patients with deleterious or suspected deleterious germline *BRCA1/2*‐mutated (gBRCAm) advanced ovarian cancer who have been treated with three or more prior lines of chemotherapy [14]. Four other PARPi, talazoparib (Pfizer) rucaparib (Clovis), niraparib (GSK) and pamiparib (BeiGene), have also been approved for the treatment of cancer by regulatory bodies [15].

Following the relative success of PARPi, a series of novel DDR inhibitors have now been discovered including inhibitors of the phospho‐inositol kinases ATR, ATM and DNA‐PK [16] and the DNA polymerase/helicase Polθ (Polymerase Theta) [17, 18]. In addition, the inhibition of other DDR proteins, such as the DNA helicase WRN [19, 20, 21], have been identified as having synthetic lethal interactions with DNA repair defects in cancer, suggesting these might also make good targets for drug discovery (Glossary). In this review, we will discuss ATR inhibitors, Polθ inhibitors and WRN inhibition as potential treatments for cancer and highlight how lessons from the discovery and development of PARPi and the study of PARPi resistance could inform the clinical development and use of new DDR inhibitors.

## 2 Resistance to PARP inhibitors

**Fig. 1:**
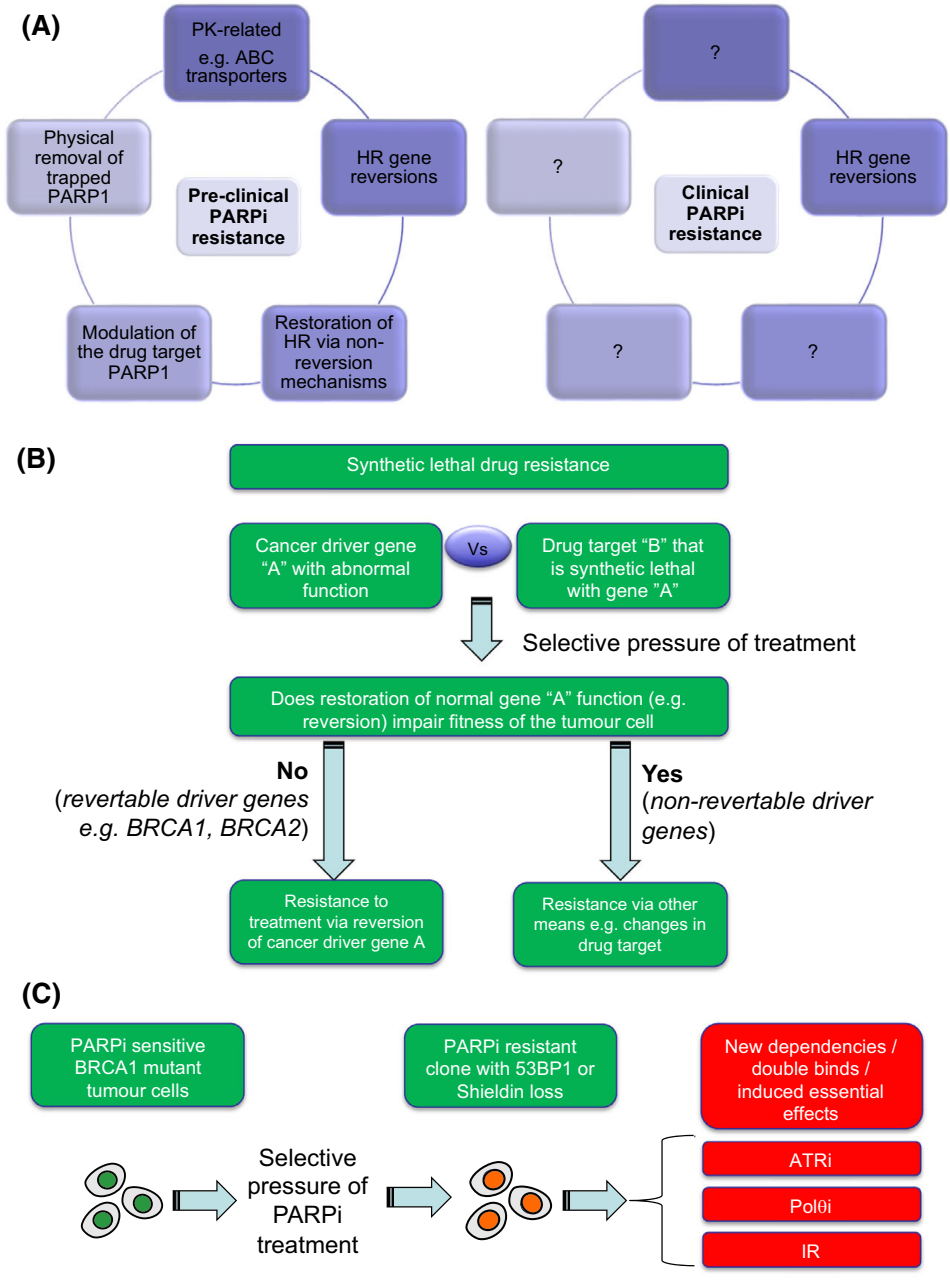
DDR inhibitor resistance. (A) In pre‐clinical models (*e.g*. tumour cell lines and genetically engineered mice, mechanisms of PARPi resistance have been identified that can be classified into four broad groups as shown on left. How many of these resistance mechanisms operate in the clinic is currently unclear. HR gene reversion mutations have been seen in multiple patients (right hand image), but do not explain all cases of PARPi resistance (see main text). Anecdotal numbers of PARPi resistant patients with either *ABC* transporter gene fusions, *53BP1* mutation or *PARP*1 mutation have been identified, but the true frequency of these mechanisms of resistance remain to be established. (B) Revertable and non‐revertable genes in synthetic lethal resistance. Synthetic lethal resistance describes the situation where drug resistance to a synthetic lethal treatment is caused by modulation of the synthetic lethal partner (*e.g*. reversion of *BRCA2*), as opposed to being caused by changes in the drug target. The potential for reversion emerging as a cause of synthetic lethal resistance must be determined by whether the reversing the dysfunction of the synthetic lethal partner (gene ‘a’) impairs the fitness of the tumour cell. The reversion of *BRCA2* has no deleterious effects on tumour cell fitness and indeed reversion gives a tumour cell a fitness advantage in the face of PARPi treatment. Other driver genes, however, may not be revertable, as their continued dysfunction is essential for the tumour cell to survive (*e.g*. addicted oncogenes). For synthetic lethal interactions involving these non‐revertable genes, other forms of drug resistance might predominate, including alterations in the drug target itself. (C) New therapeutic vulnerabilities caused by drug resistance mechanisms. In some cases, the mechanism of cancer drug resistance that emerges upon treatment creates new therapeutic vulnerabilities, not previously present or as profound in the pre‐treated state. For example, in pre‐clinical models, PARPi resistance in BRCA1 mutant tumour cells can be caused by loss of 53BP1 or Shieldin complex function (see main text). These mechanisms of PARPi resistance, whilst giving the tumour cell a fitness advantage in the face of PARPi treatment, also impart a fitness disadvantage in the face of either ATRi, Polθi or ionising radiation (IR) exposure, an evolutionary double bind or induced essentiality effect.

A series of PARPi resistance mechanisms have been identified, mainly through preclinical studies using *BRCA1/2*‐mutant tumour cells and/or mice (Fig. 1A). For example, *BRCA2* mutant tumour cell lines that become PARPi resistant after long term *in vitro* PARPi exposure develop reversion mutations (Glossary) [22], i.e. secondary mutations in *BRCA2* that compensate for the original pathogenic mutation. These reversion mutations (which also occur in *BRCA1, PALB2, RAD51C* and *RAD51D*) [23, 24, 25] restore the reading frame of the gene and thus encode functional proteins which restore HR. Reversion mutations also cause platinum salt resistance [22, 26, 27]. There are now reports of reversions in several hundred patients treated with either PARPi and/or platinum salts [25, 28] and efforts are currently underway to convert experimental methods for identifying reversions, such as DNA sequence capture and sequencing [25, 29], into clinical‐grade biomarkers. BRCA1 is also rendered inactive in some cancers via *BRCA1* promoter hypermethylation; it is likely that loss of *BRCA1* methylation during or before treatment can also result in an effective reversion of the HR phenotype [8, 30, 31, 32].

Despite the identification of reversion mutations in many PARPi‐resistant patients (some estimates suggest > 40%), there is a considerable fraction of the PARPi‐resistant patient population where the cause of PARPi resistance is not known [13]. Pre‐clinical studies have suggested that alterations in a series of other DNA repair proteins cause PARPi resistance by restoring HR without the necessity for genetic reversion of a mutant *BRCA1/2* allele. The causative proteins in these non‐reversion mechanisms include 53BP1 [33] (encoded by *TP53BP1*), RIF1 [34], SHLD1 [33, 35, 36, 37], SHLD2 [33, 35, 37, 38], SHLD3 [33, 37], REV7 [39] (encoded by *MAD2L2*), the CST complex [37, 40], PTIP [41], EZH2 [42], DYNLL1 [43, 44], SLFN11 [45]*,* amplification in *TRIP13* [46] or an increase in TIRR [34]. Thus far, extensive evidence for genetic changes in the genes encoding these proteins in clinical PARPi resistance is limited, although *TP53BP1* mutation has been reported in a case of acquired resistance [47]. This may be due, at least in part, to the absence of these genes on common ctDNA sequencing panels used to profile liquid biopsies in patients with drug resistance.

In addition to the restoration of HR (either by reversion or via the non‐reversion mechanisms described above) changes in the drug target can also cause PARPi resistance in pre‐clinical models of cancer. The primary target of clinically‐used PARPi is PARP1 [48, 49]. This DNA‐associated protein is activated by binding to damaged DNA and uses NAD+ to synthesise Poly (ADP‐ribose) chains (PAR) on adjacent substrate proteins (PARylation) and itself (autoPARylation). As well as inhibiting this catalytic activity, each of the clinically‐approved PARPi also alters the conformation of PARP1 so that its normal release from damaged DNA is impaired, a phenomenon known as PARP1 trapping (Glossary) [48, 49]. Mutations in *PARP1* that prevent PARP1 trapping cause profound PARPi resistance in pre‐clinical *in vitro* and *in vivo* models of *BRCA1* mutant cancer and have been seen in a single case of clinical PARPi resistance [50]. Some of these PARP1 resistance‐causing mutations sit within the DNA binding ZnF domains of PARP1, but others are located elsewhere in the protein, including the WGR domain, that sits between the ZnF and the catalytic domain of the protein [50]. Part of the normal removal of PARP1 from chromatin also involves PARP1 autoPARylation, an activity that is opposed by the catalytic activity of Poly (ADP‐Ribose) glycohydrolase, PARG, which hydrolyses ribose‐ribose bonds in PAR. Loss of PARG also causes PARPi resistance [51], likely by altering the amount of trapped PARP1. Other mechanisms of removal of trapped PARP1 from DNA also operate, including the p97 segregase pathway [52].

A final category of resistance mechanisms to PARP inhibitors is via upregulation of drug efflux pumps that reduce the amount of PARPi in the cell. This has been shown in genetically modified mouse models, where *Abcb1a/b* upregulation is seen in *Brca1;p53* (KB1P) mammary tumours that develop resistance to PARPi [53]. Deleting the genes encoding these transporters, or using alternative PARPi that are not Abcb1 substrates, delays resistance in these models and allows other resistance mechanisms such as 53BP1 loss to emerge [54]. *ABCB1* gene fusions have been observed in treatment‐refractory breast and ovarian cancers, and therefore may also be a source of clinical resistance to PARPi that are a substrate for this pump [55, 56].

Based on much of the work described above, a classification of different forms of PARPi resistance is starting to emerge (Fig. 1A). For example, one could classify the mechanisms into: (a) reversion‐based mechanisms that restore HR gene function; (b) non‐reversion‐based mechanisms that restore HR (e.g. loss of 53BP1 etc.); (c) modulation of the drug target (e.g. PARP1 mutation or via loss of PARG); (d) physical removal of trapped PARP1 (e.g. via the activity of p97); or (e) pharmacokinetic mechanisms that reduce the active, cellular, concentration of the drug.

## 3 Resistance to ATR inhibitors

ATR (ataxia telangiectasia and Rad3‐related) is one of the apical kinases of the DNA damage response. The ATR kinase complex is critical for recognising and triggering a response to replication stress (RS) (Glossary), a collection of phenotypes that describe abnormal replication fork function e.g. fork slowing, stalling, collapse or an increase in replication fork speed [57]. RS is common in cancer; for example, the increase in replication that results from oncogene activation (e.g. via Myc or Cyclin E upregulation) is a well‐established cause of RS [1, 58, 59]. In response to abnormal fork progression, ATR, along with its binding partner ATRIP, is recruited to the extended tracts of RPA‐coated single‐strand DNA (ssDNA) that often form at dysregulated forks [60, 61]. RPA‐bound ATR is then trans‐activated by TOPBP1 [62] or ETAA1 [63, 64], which leads ATR to phosphorylate and activate downstream effectors including the kinase CHK1 [65]. These effectors stall the cell cycle, mediate DNA repair, prevent apoptosis and limit the firing of latent replication origins, which could otherwise exacerbate RS [65]. In totality, this ATR‐mediated RS response allows cells to repair and restart replication forks so that replication can be completed before DNA is divided between daughter cells. When ATR is partially inhibited, which can be achieved via drug‐like small molecule kinase inhibitors (ATRi), the normal response to RS is impaired [66]. ATRi elicit anti‐tumour effects in both pre‐clinical cancer model systems [67] and in early phase clinical trials [68], without eliciting severe, non‐tumour toxicity; this is presumably because ATR inhibition exacerbates pre‐existing tumour cell‐specific RS to the point where tumour cells are not viable.

Multiple, highly selective ATRi are currently in clinical development including AZD6738/ceralasertib (AstraZeneca) [69], BAY1895344/elimusertib (Bayer) [70], M6620/VX970/berzosertib (Vertex/Merck KGaA) [71, 72], M4344/VX‐803/gartisertib (Vertex/Merck KGaA) [73], RP‐3500 (Repare) [74], ART0380 (Artios). These ATRi are being investigated both as monotherapies [68, 73, 75, 76] as well as in combination with classical chemotherapies [68, 77], targeted therapies including PARPi [75], radiotherapy [78] and many others [79].

Multiple cancer‐related ATR synthetic lethal effects have been described that, at least in part, provide the rationale for the clinical use of ATRi in biomarker‐defined subsets of patients. For example, ATR is synthetic lethal with the closely related PIKK family kinase ATM [80, 81, 82, 83, 84], suggesting ATRi could serve some utility in the treatment of cancers, such as gastric cancer, where ATM is defective [85]. Indeed, deleterious *ATM* mutations and/or loss of protein have been associated with antitumour responses to ATRi in an early phase clinical trial [76]. Whilst ATR and ATM primarily recognise distinct forms of DNA damage (replication‐associated damage *vs*. non‐replication associated double‐strand DNA breaks, respectively), there is considerable overlap in downstream effectors and extensive crosstalk between these two signalling pathways [86]. It is likely that each kinase can partially compensate/buffer for the loss of the other, whilst simultaneous loss is simply not tolerated by cells.

Amplification and overexpression of oncogenes such as *Cyclin E (CCNE1)* [87, 88, 89], *Ras* [90], *Myc* [91, 92] and *CDC25A* [88] have also been linked with increased replication stress and enhanced sensitivity to ATRi, whereas genetic screens have identified a range of additional cancer‐associated ATRi‐related synthetic lethal effects including those with *ARID1A* [93], *RNASEH2A/RNASEH2B* [94], *POLE3/POLE4* [95], *APOBEC3A/APOBEC3B* [96] amongst others [97, 98, 99, 100, 101, 102, 103, 104, 105]. To what extent these pre‐clinical observations translate to clinically meaningful biomarkers remains to be assessed in clinical trials [75]**.**

As ATRi have only recently entered clinical development, most of what is understood about ATRi resistance comes from pre‐clinical studies. For example, pre‐clinical genetic perturbation screens have indicated that mutation or loss of *Cyclin E*, *CDK2* or *Myc* can cause ATRi resistance [94, 95, 104]. Additional resistance mechanisms also point to stalling of the cell cycle as a resistance mechanism, for example via loss of CDC25A/B phosphatase activity [94, 95, 106, 107], or via loss of the CDK8/Cyclin C complex [94, 95, 108]. Similarly, mutation of the pro‐mitotic transcription factor FOXM1 has been linked to ATRi resistance [104]. This observation is consistent with work showing that the S/G_2_ cell cycle transition is controlled by a CDK1‐directed FOXM1 phosphorylation switch that is blocked by ATR until S phase has successfully been completed [109]. Mutations in ECT2, a Rho GTPase exchange factor linked to the DDR have also been identified as a cause of ATRi resistance [106, 110]. As yet, these mechanisms of ATRi resistance have not been validated in clinical trials, but in principle could now be assessed.

Based on knowledge of PARPi resistance (and many other drug classes), one could speculate that a likely route to ATRi resistance would be through mutations of the target gene itself, *ATR*. For example, kinase mutations that sterically hinder access of small molecule inhibitors to kinase catalytic domains, whilst allowing catalytic activity, cause resistance to other targeted kinase inhibitors [111, 112, 113, 114]. If such mutations do drive ATRi resistance, with careful drug design, it may be possible to identify second generation ATRi that overcome this form of resistance [115, 116].

PARPi resistance can also be caused by reversion of a synthetic lethal partner gene (e.g. *BRCA2*). Is it possible that a similar mechanism could also drive ATRi resistance? This may depend upon the specific cancer driver gene that is synthetic lethal targeted by ATRi. For example, in PARPi resistance, reversion of *BRCA1, BRCA2, PALB2, RAD51C*, or *RAD51D* occur because the continued fitness of tumour cells is not dependent upon the continued dysfunction of these genes; tumour cells with reversions are clearly no longer reliant or dependent upon defective HR for their survival (Fig. 1B). These particular tumour suppressors likely have a “Pandora Box” effect, (Glossary) where their dysfunction fosters mutagenesis by enabling mutation, perhaps for a defined period of time. Beyond this mutagenic period, their status, functional or dysfunctional, does not appear to have a large impact on tumour cell fitness, other than possibly the evolvability of the cell when faced with a new selective pressure (Fig. 1B). It is not clear whether the cancer driver genes that are synthetic lethal with ATRi have the same “Pandora Box” effects as HR‐controlling tumour suppressors*,* or whether their continued dysfunction is key to the fitness of tumour cells. For example, it is possible that a permanent SWI/SNF defect caused by *ARID1A* mutation (synthetic lethal with ATRi [93]) is required for the transcriptional programme that maintains the fitness of ovarian clear cell carcinomas [117, 118]. If this is the case, unless some other alteration in the tumour cell can compensate for the restoration of ARID1A function, then it is unlikely that reversion of an *ARID1A* mutation is likely to be tolerated by the tumour cell, making it unlikely that reversion of this gene emerges as a cause of ATRi resistance. We therefore foresee a future where cancer driver genes are defined as “revertable” or “non‐revertable” based on how drug resistance emerges, information that possibly indicates whether the continued dysfunction of the gene is still required by the tumour cell. This, in turn, may inform which cancer genotypes are likely to respond well to ATRi and which are likely to acquire resistance through reversion or adaptation.

ATR inhibitors clearly elicit multiple phenotypes that commensurate with the multiple substrates and downstream processes that ATR controls [119] and so some consideration must be given to which of these processes (or indeed which combinations of these processes) must be reversed to mediate the maximal level of ATRi resistance. Cell‐based genetic screens, which normally involve exposing cells to ATRi *in vitro* for a relatively limited time, suggest profound ATRi resistance can be achieved by pausing the cell cycle at S/G_2_ or G_2_/M (i.e. after S but before mitosis), so that the capacity to repair the effects of replication fork stress is maximised [104, 107]. Whether such mechanisms mediate profound ATRi in a more clinical setting, where ATRi treatment continues over months, not days, remains to be seen. It is possible, for example, that when viewed from the perspective of this much longer treating time, that only a combination of suppressing the DNA damage that ATRi cause, together with pausing of the cell cycle to enable repair, mediates profound ATRi resistance in cancer. The observation that pausing the cell cycle at S/G_2_ or G_2_/M can cause ATRi resistance might also suggest that some consideration might be given to thinking about how combination therapy is used with ATRi; it seems reasonable to think that other cancer drugs (such as CDK4/6 inhibitors) that work by reimposing key cell cycle checkpoints in tumour cells might be antagonistic to the effects of ATRi.

## 4 Resistance to Polθ inhibitors

Polθ is an A‐family DNA polymerase and helicase with key roles in theta‐mediated end joining (TMEJ, also known as alt‐NHEJ or microhomology‐mediated end joining, MMEJ), base excision repair (BER) and translesion synthesis (TLS) (Glossary) [120, 121, 122, 123, 124]. TMEJ is one of five distinct repair processes that repair DNA double‐strand breaks (DSB), the others being non‐homologous end joining (NHEJ), HR, single strand annealing (SSA) and break‐induced replication (BIR). Of these, NHEJ preferentially repairs unresected DSB ends [125, 126, 127], whereas HR and TMEJ require DNA resection to generate a 3′ ssDNA overhang [128, 129, 130]. As described earlier, HR is a conservative, template‐dependent DNA repair process involving BRCA1 and BRCA2, repairing DNA damage using strand invasion into a homologous chromosome or sister chromatid followed by templated DNA synthesis. In cells lacking HR, such as *BRCA*1/2 deficient cancer cells, TMEJ serves as an essential backup mechanism that still allows resected DSBs to be repaired [131] through the activity of PARP1, DNA ligase III and Polθ (encoded by *POLQ*) [132].

The interest in Polθ as a therapeutic target in cancer was highlighted by the observations that Polθ confers resistance to the topoisomerase inhibitors etoposide and camptothecin [133, 134], ATRi [133] and ionising radiation (IR) [135, 136, 137]. Furthermore, genetic inactivation of *POLQ* is synthetic lethal with HR defects caused by either *BRCA1, BRCA2*, *ATM, RAD51C* or *FANCD2* defects [138, 139, 140, 141, 142, 143, 144, 145] and tumour overexpression of *POLQ* correlates with HRD status and a poor clinical outcome [138, 146, 147]. Recently, some of these HR‐related synthetic lethal effects have been recapitulated with novel small molecule inhibitors that target Polθ’s DNA polymerase [17] or helicase functions [18]. These Polθ inhibitors not only target *BRCA1/2* mutant tumour cells and enhance the synthetic lethal effects of PARPi, but also target PARPi resistant tumour cells with defects in the 53BP1/Shieldin [17, 18], described above.

Although very little is known about how resistance to Polθi might emerge, it has already been shown that pre‐existing *BRCA2* reversion mutations that cause platinum‐salt and PARPi also cause resistance to Polθi [17, 18]. Interestingly, these observations provide the key evidence that the *BRCA2* defect in BRCA2‐mutant tumour cells is the primary driver of Polθi sensitivity, and not some downstream consequence of *BRCA2* mutation, such as genomic mutations elsewhere in the genome that have arisen because of the HR defect in these cells. Combined with the recent data suggesting that 53BP1‐pathway defects in *BRCA1* mutant, PARPi resistant tumour cells cause profound sensitivity to Polθi [17, 18] it also suggests that if Polθ inhibitors are to be used when PARPi or platinum resistance has occurred, this should be in those with 53BP1/Shieldin defects, and perhaps not in those where the dominant tumour clone has a *BRCA1/2* reversion.

The issue of *BRCA1/2* reversion also highlights another feature of Polθ biology that might reflect a slightly different utility for Polθi compared to other agents that target defective HR. TMEJ, one of the DNA repair processes that Polθ controls, has itself been implicated in the formation of reversion mutations in *BRCA1*, *BRCA2* [22, 27] and *PALB2* [23, 148]. For example, DNA sequence analysis of reversion mutations highlights that many of these are deletions flanked by tandem DNA repeat sequence microhomologies, a feature indicative of TMEJ operating in the absence of HR [25, 28]. Although pre‐existing reversion mutations cause Polθi resistance [17, 18], the potential to prevent new TMEJ‐mediated reversions via Polθ inhibition could be widely exploited by using these agents either before or even in combination with PARPi or platinum salts [17]. This is not to say drug resistance would not emerge in this setting, but the possibility of targeting TMEJ to suppress reversion formation may at least drive resistance to emerge in forms that are perhaps simpler to treat than revertant cancers, which are not currently treatable with a targeted approach.

What other processes might emerge that could cause clinical Polθi resistance? As described above, mutations in the drug target itself might cause resistance – for example missense or in frame deletions that allow Polθ activity in the presence of small molecule inhibitor. In addition, pharmacokinetic (PK) resistance mechanisms (such as increased drug metabolism or cellular export) could play a part; countering this, the judicious selection of clinical Polθ inhibitors with properties that make such PK issues a likely driver of resistance seem already to be used (Glossary) [17]. Alternatively, changes in pathways that compensate for the loss of Polθ activity could conceivably cause resistance, as could molecular changes that compensate for dysfunction in the tumour suppressor being synthetic lethal targeted by a Polθi (e.g. *BRCA1, BRCA2*). What these changes might be remains to be determined, however we note that in tumour cells with both *BRCA1* and *53BP1* defects (where Polθi synthetic lethality is relatively profound), for the full degree of Polθi resistance to emerge, the dysfunction in BRCA1 *and* 53BP1 may have to be compensated for (as opposed to just a compensatory change in BRCA1 *or* 53BP1). This would potentially make profound Polθi resistance less likely in a BRCA1/53BP1‐defective setting than in, for example, a BRCA1 defective setting, where only a single compensatory change might be required. As the ability to predict how cells with a particular molecular make‐up (e.g. combined BRCA1/53BP1 defects) rewire in the face of a particular perturbation (e.g. Polθ inhibition) is in its infancy, only further experimentation will confirm whether a synthetic lethality that targets two combined cancer‐associated defects is less prone to resistance than those that target one defect.

## 5 Resistance to WRN inhibition

Werner syndrome ATP‐dependent helicase (WRN) is a member of the RECQ family of DNA helicases, involved in unwinding of double‐stranded DNA for replication and repair processes [149]. WRN is notable in that it is the only member with 3′ to 5′ exonuclease activity in addition to its helicase activity [150]. Individuals carrying germline mutations in the *WRN* gene exhibit characteristics of Werner syndrome [151], including genomic instability [152, 153], cancer predisposition [154] and accelerated ageing [155]. WRN helicase is of interest from a cancer therapy perspective in that it is observed to be highly expressed in rapidly dividing cells [156] and cancer [157]. Whilst induced loss is generally well‐tolerated in healthy cells, targeting WRN can induce sensitivity to DNA damage [158, 159]. Importantly, multiple studies have identified WRN to be a genetic dependency/synthetic lethal target in cancers with microsatellite instability (MSI) [19, 20, 21], raising the possibility of selective targeting of this subset of cancers by WRN small molecule inhibition [160].

MSI arises in cancer with the loss of DNA mismatch repair (MMR) pathways, often through mutation or epigenetic silencing of *MLH1* or *MSH2* tumour suppressor genes [161]. MMR is critical for detection and resolution of spontaneous DNA replication errors and as such, MMR inactivation leads to a hypermutator phenotype and genomic instability [162]. Of particular relevance to WRN, MSI can lead to expansion of microsatellite DNA sequences (Glossary), including (TA)n repeats; this microsatellite instability often causes the formation of unusual DNA secondary structures, stalling of replication forks and activation of ATR [163]. WRN is specifically required to unwind DNA at such secondary structures; in the absence of WRN, TA repeats are cleaved by MUS81, an event which leads to chromosome shattering and cell death [163]. Sensitivity of MSI cells to WRN loss is specifically dependent on the helicase function of WRN and not its exonuclease activity [19, 20, 21].

Compared to the *BRCA1/2 vs*. PARPi synthetic lethality, the synthetic lethality between WRN inhibition and MSI is distinct; WRN inhibition does not target a DNA repair defect *per se* but targets the mutagenic consequence of a DNA repair defect, namely the presence of expanded TA‐dinucleotide repeats [163]. Indeed, MSI+ cell lines that are refractory to WRN loss, are those that have a notable absence of expanded TA repeats [164]. Conversely, PARPi synthetic lethality targets a defective DNA repair mechanism (homologous recombination when caused by *BRCA1/2* defects) but does not appear to target the mutagenic consequences of this HR defect. A sign of this is that reversion mutations in *BRCA1/2*, which restore HR without reversing the existing mutagenic consequences of the HR defect, cause PARPi resistance [22, 27]. Furthermore, when *BRCA2* defects are experimentally imposed upon cells *in vitro* or in animals, these cause PARPi sensitivity without necessarily recapitulating the mutagenic consequences of *BRCA2* mutation seen in human cancers, again suggesting it is the primary HR defect that is important to PARPi sensitivity and not how defective HR moulds the genome. For this reason, it seems unlikely that reversion of a DNA repair‐associated tumour suppressor such as *MLH1* or *MSH2* would be a likely source of resistance to WRN inhibition in the same way that *BRCA1/2* reversions cause resistance to PARPi. Given this, other mechanisms are likely to predominate. A drug‐like WRN inhibitor does not yet exist and therefore it is difficult to predict whether or how mutation or modulation of WRN itself could drive resistance. However, the existing mechanistic dissection of the MSI/WRN synthetic lethality already predicts a likely source of resistance. As described above, the MSI/WRN synthetic lethality appears to be driven by MUS81 nuclease cleavage of expanded TA‐dinucleotide repeats (along with its scaffold protein SLX4) [163]; this suggests that loss of MUS81/SLX4 function (or at least partial loss compatible with cell fitness) might drive WRN inhibitor resistance [163]. Of course, such a MUS81 defect might itself open up other therapeutic vulnerabilities (e.g. PARPi [165] or WEE1i sensitivity [166]), suggesting how such a mechanism of resistance, were it to occur, could be targeted. This is discussed in Section 6, below.

## 6 Targeting DDR inhibitor resistance

There are many reasons for understanding how drug resistance in cancer emerges. This information can be used to inform the identity of biomarkers that allow patient stratification for effective treatment and to avoid the use of treatments that are ineffective. In addition, understanding how drug resistance emerges is the first step towards devising strategies that enhance the overall effectiveness of treatment. For example, identifying and understanding a mechanism of drug resistance could lead to the design of combination therapies that target a primary defect in a cancer alongside the resistance mechanism to the first treatment. Identifying mechanisms of drug resistance could also inform the sequential use of treatments, where the first targets a primary defect in a cancer whilst the second targets the mechanism of resistance that emerges in response to the first treatment.

In this respect, one lesson the explosion in identifying synthetic lethal interactions in cancer has indicated is that if one is able to identify a molecular change in a tumour cell (for example a change in a cancer driver gene), then there is the possibility of identifying a synthetic lethal interaction that targets this cancer‐specific change. The same is probably true of cancer drug resistance mechanisms involving DDR inhibitors; when a mechanism of resistance emerges, a new set of vulnerabilities also emerge (Fig. 1C). This is in essence a form of induced essentiality, where an adaptation to one selective pressure causes a new essentiality, reflecting the homeostatic changes that occur in response to the original adaptation [167]. Alternatively, this could be viewed as an evolutionary double‐bind, where a drug resistance adaptation drives the tumour cell population down an evolutionary route in which a new vulnerability emerges [168]. For example, a 53BP1 defect that emerges to homeostatically enable *BRCA1* mutant cells to survive the selective pressure of PARPi treatment, imposes ionising radiation (IR) [169], ATRi [170] or Polθ inhibitor [17, 18] hypersensitivity upon cells (Fig. 1C).

The success of this strategy is of course dependent upon whether a resistant tumour is clonal. Parallel evolution of multiple different resistant clones, each with a different mechanism of resistance (and thus different synthetic lethal effects), could present a challenge to this approach. However, improvements in the detection of early emerging drug resistant clones (e.g. via the use of cfDNA profiling) [171] could allow treatment to be rapidly adapted so that dominant, drug resistant, clones can be targeted relatively soon after these emerge. Ideally, this targeting of drug resistant clones would be made before highly heterogeneous populations of drug resistant clones, each with a different mechanism of resistance (and thus requiring a different therapeutic approach), emerge. Finally, for reasons we explain earlier, targeting the consequences of DDR defects, (e.g. their mutational consequences such as microsatellite expansion) might turn out to be a more robust therapeutic approach than targeting the primary DDR defect itself, given it is difficult to understand how the mutational consequences of many DDR defects could be easily reversed. Whilst this might be theoretically possible with WRN inhibitors in cancers with expanded TA repeats, approaches that target cancers with different types of mutational signature are not known but could be identified (an attempt to identify these is described here [172]). For example, in experimental models, though a *BRCA2* reversion is able to restore HR and cause PARPi resistance, it does not restore a normal diploid genome to tumour cells; PARPi‐resistant *BRCA2*‐revertant cells retain a highly disordered genome and are still p53 mutant [173]. Perhaps unsurprisingly, these revertant cells also retain ATR or WEE1 inhibitor sensitivity [173], which is possibly driven by the disordered genome/mutant p53 status of revertant cells. Identifying vulnerabilities that are associated with distinct types of disordered tumour genome might therefore provide additional therapeutic routes going forward.

## 7 Glossary

**Homologous recombination (HR)**. A form of genetic recombination. HR involves the exchange of genetic information between two similar or identical (i.e. homologous) nucleic acid sequences, such as DNA sequences on sister chromatids or homologous chromosomes. HR is often used to faithfully repair double strand DNA breaks, with the homologous DNA sequence being used as a DNA template upon which newly synthesised DNA is generated.

**Nucleotide Excision Repair (NER).** A form of DNA repair which repairs single stranded DNA (ssDNA) damage.

**Platinum‐based chemotherapy.** Chemotherapeutic agents used in cancer treatment that are salts of platinum. Drugs in this class include cisplatin, carboplatin and oxaliplatin. Platinum salts work by causing the formation of crosslinks within DNA double helices and/or by causing crosslinks between DNA and proteins. These crosslinks are thought to impair the fitness of cells by preventing transcription and translation.

**Bevacizumab.** A recombinant monoclonal antibody treatment for cancer that inhibits Vascular Endothelial Growth Factor A.

**Synthetic lethality.** A form of genetic interaction. Two genes (or proteins) are said to be synthetic lethal or involved in a synthetic lethality when inhibition of either gene is compatible with cell viability, but where inhibition of both genes is not. Genes involved in a synthetic lethal relationship are often termed synthetic lethal partners.

**Reversion mutations.** Mutations that restore (“revert”) the function of a mutated gene from a dysfunctional form to a functional form.

**PARP1 trapping**. An effect where PARP inhibitors not only inhibit the catalytic activity of PARP1 but also increase the amount of PARP1 bound to DNA (or in the chromatin fraction of cells).

**Replication stress.** Molecular processes that lead to the abnormal behaviour of the replication fork. Replication stress often manifests as an extreme increase or decrease in replication speed, or in the stalling or collapse of replication forks.

**Pandora Box effect.** A biological effect that is irreversible. Named after Pandora, a character from Greek mythology. For example, the mutations in the genome caused by BRCA1 or BRCA2 dysfunction are thought to persist even when BRCA1 or BRCA2 function is restored by reversion mutations.

**Theta‐mediated end joining (TMEJ)**. Also known as Microhomology Mediated End Joining or Alt‐NHEJ. A form of double strand DNA break (DSB) repair which involves resection (cutting back) of DNA at the DSB, alignment of small regions of identical (microhomologous) sequences close the ends of the resected DNA and ligation of DNA ends at the point of microhomology, with the result that genomic DNA inbetween the regions of microhomology is deleted. This process is mediated by DNA Polymerase Theta (Polq), hence the name.

**Pharmacokinetic (PK) resistance mechanisms.** Mechanisms of drug resistance caused by a change in the pharmacokinetics of the drug involved. For example, via increased metabolic degradation of the drug or by an increase in the activity of small molecule transmembrane pumps that reduce the intracellular concentration of a drug.

**Microsatellite DNA sequences.** A short segment of DNA, usually 1–6 bp in length, that is repeated multiple times in succession at a particular genomic location. For example, TA repeats (e.g. TATATA). Replication of microsatellite DNA sequences often results in the addition of new repeat sequences and the formation of DNA mismatches to newly synthesised DNA. These mismatches are often removed from newly synthesised DNA by the process of mismatch repair (MMR).

## 9 Conflict of interest

C.J.L. makes the following disclosures: receives and/or has received research funding from: AstraZeneca, Merck KGaA, Artios. Received consultancy, SAB membership or honoraria payments from: Syncona, Sun Pharma, Gerson Lehrman Group, Merck KGaA, Vertex, AstraZeneca, Tango, 3rd Rock, Ono Pharma, Artios, Abingworth, Tesselate, Dark Blue Therapeutics. Has stock in: Tango, Ovibio, Enedra Tx., Hysplex, Tesselate. C.J.L., D.Z. and S.J.P. are also named inventors on patents describing the use of DNA repair inhibitors and stand to gain from their development and use as part of the ICR ‘Rewards to Inventors’ scheme.

## 10 Author contributions

CJL conceived the concept for the review with input from JSB. All authors wrote the main text. CJL and JSB constructed the figures. All authors approved the final version of the manuscript.
